# No NETs no TIME: Crosstalk between neutrophil extracellular traps and the tumor immune microenvironment

**DOI:** 10.3389/fimmu.2022.1075260

**Published:** 2022-12-23

**Authors:** Qi Fang, Antonia Margarethe Stehr, Elisabeth Naschberger, Jasmin Knopf, Martin Herrmann, Michael Stürzl

**Affiliations:** ^1^ Division of Molecular and Experimental Surgery, Translational Research Center, Department of Surgery, Friedrich-Alexander Universität (FAU) Erlangen-Nürnberg and Universitätsklinikum Erlangen, Erlangen, Germany; ^2^ Comprehensive Cancer Center Erlangen-Europäische Metropolregion Nürnberg (EMN), Universitätsklinikum Erlangen, Erlangen, Germany; ^3^ Department of Internal Medicine 3, Friedrich-Alexander Universität (FAU) Erlangen-Nürnberg and Universitätsklinikum Erlangen, Erlangen, Germany; ^4^ Deutsches Zentrum für Immuntherapie (DZI), Friedrich-Alexander Universität (FAU) Erlangen-Nürnberg and Universitätsklinikum Erlangen, Erlangen, Germany

**Keywords:** tumor microenvironment, cancer, neutrophil extracellular traps, neutrophils, macrophages, adaptive immunity, innate immunity, immunotherapy

## Abstract

The tumor immune microenvironment (TIME) controls tumorigenesis. Neutrophils are important components of TIME and control tumor progression and therapy resistance. Neutrophil extracellular traps (NETs) ejected by activated neutrophils are net-like structures composed of decondensed extracellular chromatin filaments decorated with a plethora of granules as well as cytoplasmic proteins. Many of these harbour post translational modifications. Cancer cells reportedly trigger NET formation, and conversely, NETs alter the TIME and promote tumor cell proliferation and migration. The specific interactions between NETs and TIME and the respective effects on tumor progression are still elusive. In certain tumors, a CD4^+^ T helper (Th) 2 cell-associated TIME induces NETs and exerts immunosuppressive functions *via* programmed death 1 (PD-1)/PD-L1, both associated with poorer prognosis. In other cases, NETs induce the proliferation of Th1 cells, associated with an improved prognosis in cancer. In addition, NETs can drive macrophage polarization and often rely on macrophages to promote cancer cell invasion and metastasis. In turn, macrophages can swiftly clear NETs in an immunologically silent manner. The aim of this review is to summarize the knowledge about the mutual interaction between NETs and TIME and its impact on tumor growth and therapy.

## 1 Introduction

The tumor immune microenvironment (TIME) is orchestrated by the interaction between immune and tumor cells and is shaped by various cytokines and chemokines (1–3). It plays a pivotal role in the initiation, progression, invasion, and metastasis of cancers. Tumor infiltrating immune cells can promote or inhibit tumorigenesis.

Most studies focused on the role of adaptive immune cells in cancer. Various pre-clinical and clinical models demonstrated that T lymphocytes exert an integral role in tumor immune defence. Cytotoxic CD8^+^ T cells (CTLs) are prominent components within the TIME and form a homogenous population of cytotoxic cells that secret interferon (IFN)-γ. CTLs drive anti-tumor responses and improve patient prognosis (4). In contrast, regulatory T cells (Tregs) dampen immune responses and, thus, contribute to the immune evasion of tumor cells (5). Th cells come in different flavours and form functionally different populations, such as Th1, Th2 and Th17 cells (6). Th1 cells shape the anti-tumor immunity, induce CTLs and are associated with improved prognosis. On the contrary, Th2 cells promote humoral immunity, restrain Th1 responses and are associated with poorer prognosis (6–8). Th17 cells exhibit heterogeneity in human cancer with the expression of various activated markers, cytokines and transcriptional factors leading to different prognoses of patients. It still remains a challenge to use Th17 cells as a predictor for the prognosis in human cancer (9).

**Figure 1:**
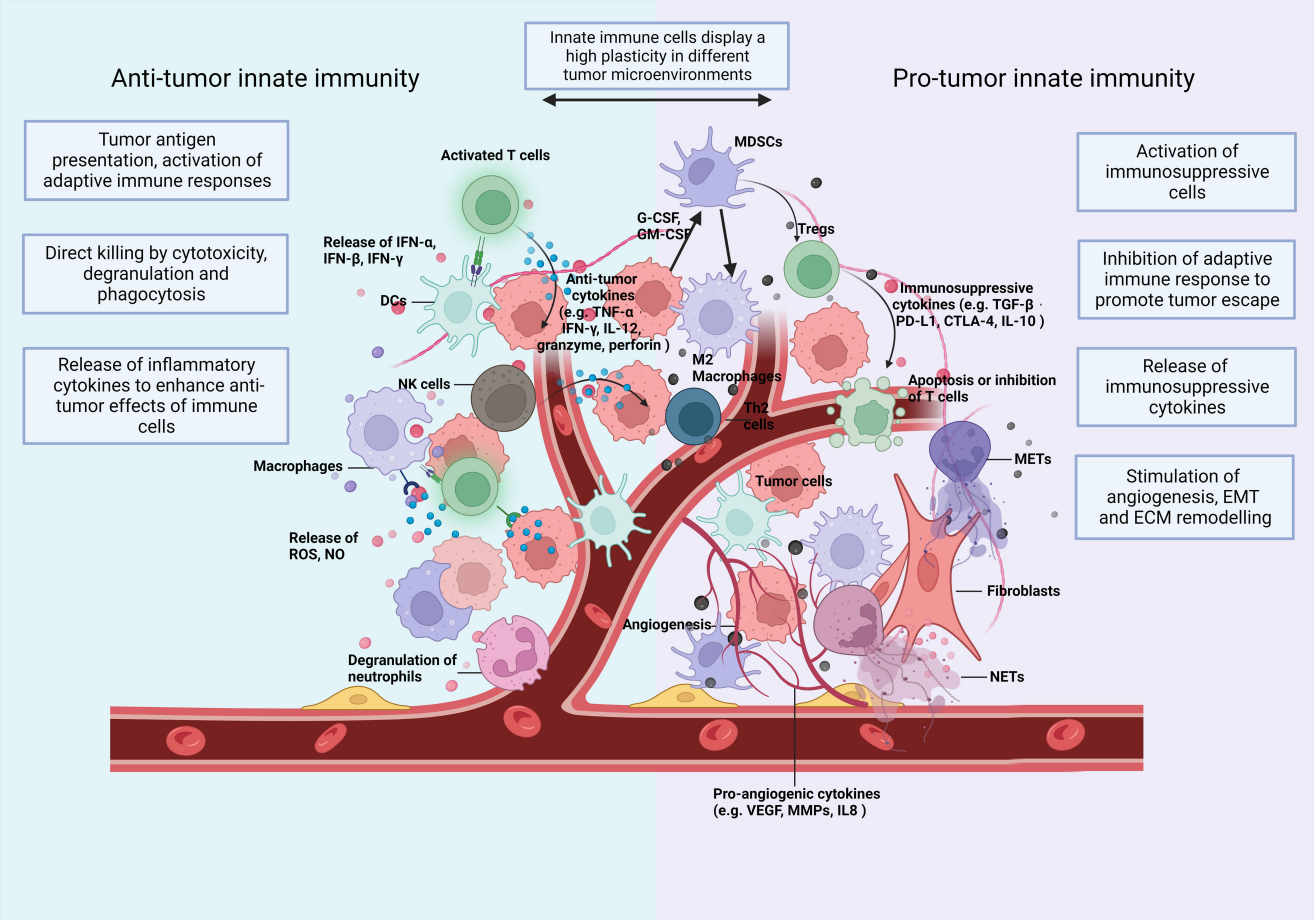
The TIME: Immune cells and soluble mediators shape the diversity of the TIME. Innate immunity plays multiple roles in cancer and shows a high plasticity depending on changes of the TIME. In Anti-tumorigenic TIMEs, innate immune cells (1) recognize and present tumor cell-derived antigenic peptides to T cells and activate the adaptive immunity (2); eliminate tumor cells directly by phagocytosis and secrete cytotoxic substances, like perforin and granzyme (3); secrete proinflammatory cytokines and, thus, expand and promote a wide variety of Anti-tumor responses. In a Pro-tumorigenic TIME, innate immune cells (1) differentiate towards an immunosuppressive phenotype with the release of immunosuppressive cytokines (2); induce the infiltration of immunosuppressive adaptive immune cells, such as Tregs and Th2 cells (3); increase the formation of extracellular traps (ETs); and (4) promote angiogenesis (5), epithelial-mesenchymal transition (EMT) and (6) extracellular matrix (ECM) remodelling. Consequently, they enhance tumor invasion and migration. The plasticity of innate immunity allows remodelling of the TIME. CTLA-4, cytotoxic T lymphocyte associated antigen 4; DCs, dendritic cells; ECM, extracellular matrix; EMT, epithelial mesenchymal transition; G-CSF, granulocyte colony stimulating factor; GM-CSF, granulocyte-macrophage colony stimulating factor; IFN, interferon; IL, interleukin; MDSC, myeloid-derived suppressor cells; METs, macrophage extracellular traps; MMPs, matrix metalloproteinases; NETs, neutrophil extracellular traps; NK, natural killing; NO, nitric oxide; PD-L1, programmed death-ligand 1; ROS, reactive oxygen species; TGF, transforming growth factor; TNF, tumor necrosis factor; VEGF, vascular endothelial growth factor.

These preclinical studies provided indispensable help for the promising improvement of the prognosis of cancer diseases over the last decades. However, in most solid tumors, particularly when CTL infiltration is low and immunosuppressive immune cell infiltration is high, metastasis is responsible for the majority of cancer-related mortality. Tumor cells may escape current immunotherapies and the resistance to immunotherapy is partly due to the dysregulation of innate immune cells (10), such as dendritic cells (DCs) (11), tumor-associated neutrophils (TANs) (12), tumor-associated macrophages (TAMs) (13), natural killer (NK) cells (14), and myeloid-derived suppressor cells (MDSCs) (15). These innate immune cells participate in the malignant progression from the primary to the metastatic tumor. They display a high plasticity often depending on the type and stage of the different tumors (**Figure 1**) (16, 17).

Neutrophils are the first line of defence against various kinds of pathogens. They primarily act as innate effector cells and account for around 70% of circulating leukocytes in humans (18). In both infections and cancer, their functions are predominantly implemented *via* degranulation, phagocytosis and the formation of neutrophil extracellular traps (NETs) (19). NETs have originally been described in 2004 as a nucleic acid based structure involved in bacterial defence (20). NETs ejected by activated neutrophils are net-like structures composed of decondensed extracellular chromatin filaments decorated with granular proteins, such as neutrophil elastase (NE), cathepsin G, myeloperoxidase (MPO), matrix metalloproteinases 9 (MMP9) and histones; the latter are often posttranslationally citrullinated (21). NETs were first identified as contributors to the innate immune response, capable of directly immobilizing and killing pathogens or releasing anti-microbial agents (20). Recently, NETs have been reported to play an important role in cancer initiation and progression (22–24). They are essential in the development of pre-metastatic niches, awakening of dormant metastases and may directly promote tumor growth by associated proteases, such as NE and MMP9 through proteolytic remodelling of laminin (12). NETs can also entrap circulating tumor cells and act as adhesion substrate to promote their adhesion, invasion and migration (25, 26). In comparison to the direct impact on cancer cells, only little is known about the mutual interaction of NETs with infiltrating immune cells in the TIME.

Macrophages are large phagocytic cells, not only pivotal for host defence, but also essential for tissue homeostasis (27). The major function of macrophages is to recognize and phagocytose cellular debris and opsonize immune complexes. Macrophages prey proteins, process them and present the respective peptides to T cells and elicit adaptive immune responses. Macrophages are regarded as one of the most important bridges between innate and adaptive immunity. Within innate immune cells, monocyte derived macrophages reflect the Th1/Th2 paradigm through their ability to differentiate into the inflammatory M1 or immune suppressive M2 phenotype *in vitro* (28). However, several other macrophage subtypes have been described in addition to M1 and M2 representing extremes of a multidimensional/spectral continuum (29). High macrophage infiltration in most solid tumors correlates with poor overall survival. It is associated with changes in cancer-related inflammation, angiogenesis, extracellular matrix (ECM) remodelling, and epithelial mesenchymal transition (EMT) of cancer cells (30–33). Macrophages can also form extracellular traps (ETs), referred to as macrophage extracellular traps (METs) (34). METs can activate the migration and invasion of tumor cells and are an independent risk factor for the prognosis of colorectal cancer (CRC) (35).

Although some studies have shown the biological function of NETs in cancer, the crosstalk between NETs, macrophages, and METs still remains elusive. The contribution of NETs to TIME including innate and adaptive immunity is also underexplored. More effective strategies may be inspired by better understanding of how the TIME and NETs interact. Therefore, this review will integrate the available knowledge in this context aiming to explore the interaction between NETs and TIME on tumor progression.

## 2 Mechanisms of NET Formation

**Figure 2:**
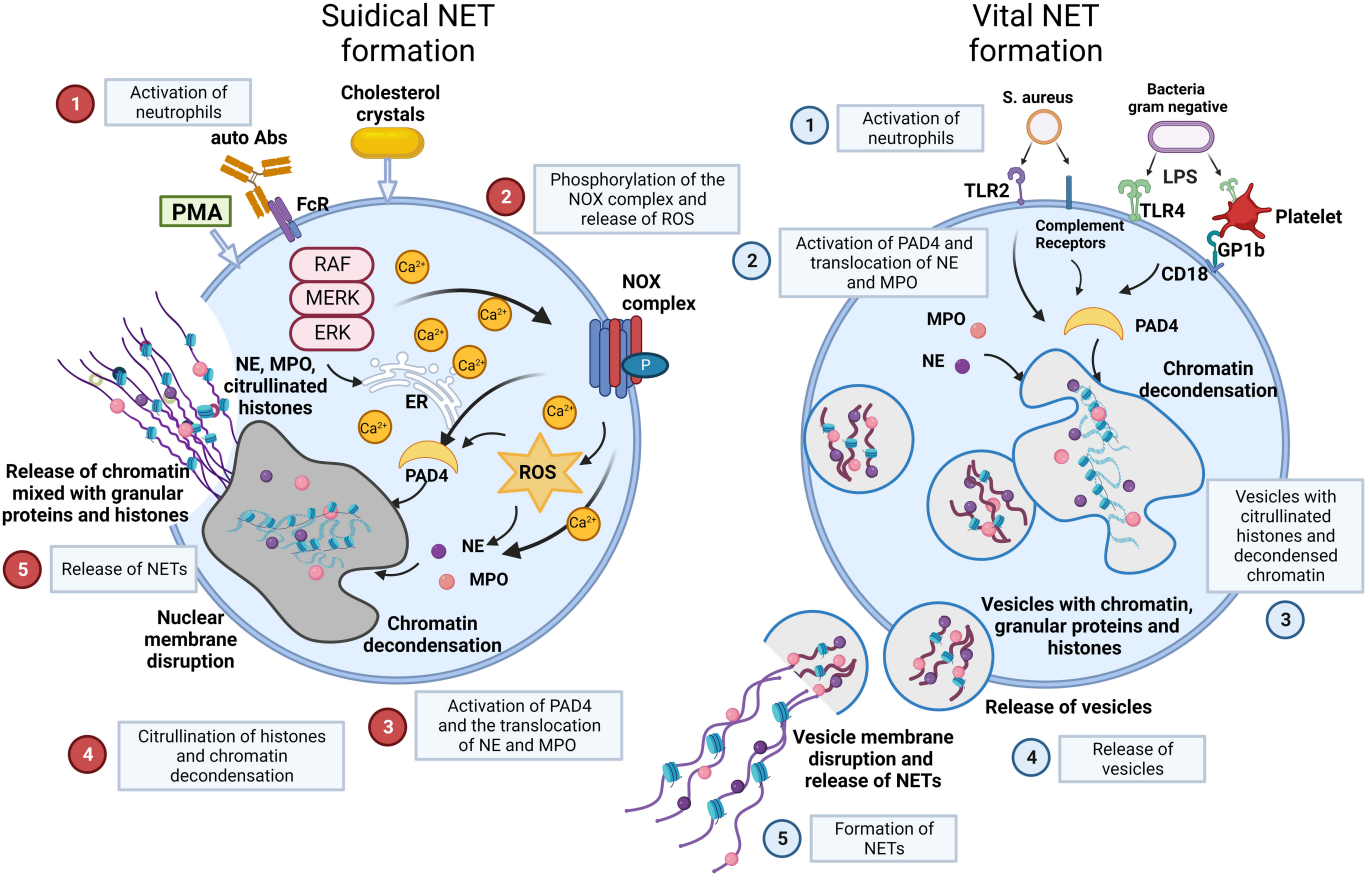
NET formation: suicidal NETosis and vital NET formation establish two main types of chromatin release by neutrophils. Suicidal NETosis is characterized by ROS generation and rupture of neutrophils. Neutrophils are activated by stimuli, such as PMA, cholesterol crystals, certain autoantibodies or immune complexes. These extracellular signals induce the phosphorylation of the NOX complex and the release of ROS. This process depends on a high Ca^2+^ concentration. Subsequently, PAD4 is activated and causes the translocation of NE and MPO from azurophilic granules to the nucleus. NE and MPO combined with PAD4 result in the citrullination of histones and chromatin decondensation. After rupture of the nuclear membrane, the decondensed chromatin enters the cytoplasm mixed with granular proteins. Finally, the cytoplasma membrane gets leaky, the modified chromatin is released from neutrophils and forms NETs. In contrast, Vital NET formation is executed in a shorter time after activation of neutrophils and can also occur in the absence of the NOX complex and ROS. Vital NET formation is initiated by stimuli, such as S. aureus through TLR2 and complement receptors, or LPS from gram negative bacteria through TLR4 or indirectly through TLR4-activated platelets. PAD4 is activated and NE and MPO translocate to the nucleus to promote chromatin decondensation. The decondensed chromatin decorated with granular proteins and histones is packed in vesicles that bud from nuclei. Subsequently, these vesicles are expelled from intact neutrophils and form NETs in the vicinity of the neutrophils. In consequence, neutrophils stay intact and can exert further functions, such as phagocytosis. Figure adopted with modifications from [36], with permission from Springer Nature, Nature medicine ^©^ [2017]. Abs, antibodies; FcR, Fc receptor; GP, glycoprotein; MPO, myeloperoxidase; NE, neutrophil elastase; NOX, NADPH oxidase; P, phosphorylation; PAD4, protein-arginine deiminase 4; PMA, phorbol-12-myristate-13-acetate; ROS, reactive oxygen species; S.aureus, staphylococcus aureus; TLR, toll like receptor.

There are two main models of NET formation: suicidal NETosis and vital NET formation (**Figure 2**) (36–38).

### 2.1 Suicidal NETosis

Suicidal NETosis begins with the activation of neutrophils by stimuli, such as immune complexes, certain autoantibodies, calcium-salt or cholesterol crystals, or phorbol-12-myristate-13-acetate (PMA) (39). These stimuli activate the NADPH oxidase (NOX) complex and lead to subsequent formation of reactive oxygen species (ROS) through Raf/MEK/ERK signalling, along with an increase in cytosolic Ca^2+^ concentrations. Then, NOX and ROS complexes induce the translocation of NE and MPO from neutrophil granules into the nucleus together with the activation of protein-arginine deiminase 4 (PAD4), which reduces positive charges from histones. This causes chromatin decondensation in the nuclei of neutrophils (39, 40). Decondensed chromatin enters the cytoplasm, mixes with granular and cytosolic proteins and is finally expelled outside neutrophils accompanied by cellular lysis. Here, it forms NETs. Suicidal NETosis causes the death of the respective cell due to membrane disintegration and this process can take several hours to complete (41).

### 2.2 Vital NET formation

Vital NET formation occurs independently of cell death in the absence of membrane disruption within minutes after stimulation of neutrophils (42). The process is initiated by stimuli such as S. aureus or lipopolysaccharide (LPS) from gram negative bacteria through toll-like receptors (TLRs) and complement receptors (CR) (36). Vital NET formation does not rely on the NOX complex or ROS. The release of nuclear DNA in vital NET formation is associated with characteristic morphological changes (1): nuclear envelope growth and the release of vesicles (2); nuclear decondensation, and (3) nuclear envelope disruption (43–45). Vital NET formation is observed more often in infectious than in non-infectious diseases. This is supported by the observation that neutrophils stay alive and are still able to perform anti-microbial functions such as chemotaxis, phagocytosis, and killing of bacteria (46). In a specific type of vital NET formation, mitochondrial DNA may also be released, and this is dependent on ROS. This process results in NET formation from 80% of the neutrophils within 15 min following stimulation with C5a or LPS (47, 48).

### 2.3 Essential factors of NET formation

Regardless of the type of NET formation certain factors, such as PAD4, NE and MPO are commonly involved in NET formation (49). However, not all of them are strictly required. PAD4 is a calcium-dependent enzyme dispersed in the nucleus, cytoplasm, and secretory granules of neutrophils. Nuclear PAD4 converts arginine in proteins (e.g. in histones H3, H2A, and H4) to citrulline. Every citrullination neutralizes one positive charge of histones and decreases their affinity for nucleic acids and concomitantly supports chromatin decondensation (50). In naive neutrophils, NE and MPO are stored in azurophilic granules (51, 52). In activated neutrophils, NE enters the nucleus, where it clips the tails of certain histones further supporting chromatin decondensation (20). Although MPO has only a minor effect on chromatin decondensation on its own, it binds to DNA and catalyzes oxidative reactions that promote NE relocation (40). Thus, MPO synergizes with NE in chromatin decondensation. Furthermore, both NE and MPO reportedly decorate the DNA backbone of NET fibers (20).

**Figure 3:**
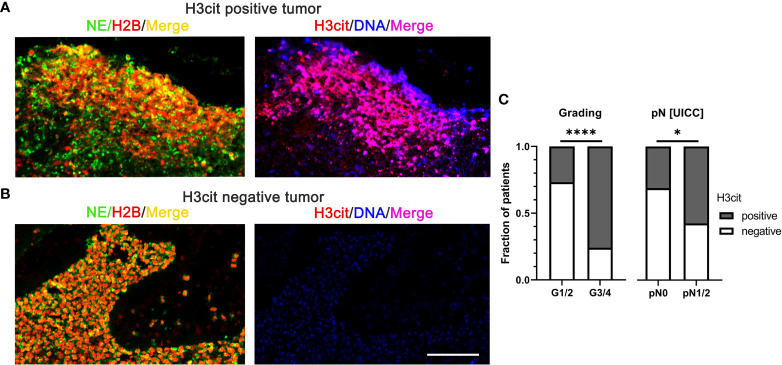
NETs in colorectal cancer identified by interstitial H3cit are associated with high histopathological tumor grades and lymph node metastasis in human CRC. **(A, B)** NE, H2B, and H3cit were used to detect NET formation on consecutive sections of CRC by immunofluorescence. Draq5 served as counterstain. Notably, regions with extranuclear H3cit colocalize with NE H2B in consecutive sections. Extracellular DNA detected by anti-DNA antibody is restricted to H3cit positive tissues. Scale bar: 75µm. **(C)** In human CRC tissues, NETs identified by H3cit positively correlated with high histopathological grading and lymph node metastasis. Results taken from [55], ^©^ [2022] Pathological Society of Great Britain and Ireland, first published by John Wiley & Sons Ltd. H2B, histone H2B; H3cit, citrullinated histone 3; NE, neutrophil elastase; NETs, neutrophil extracellular traps. ****p < 0.0001, *p < 0.05.

Histone citrullination is a characteristic feature of NET formation and the detection of citrullinated histones on extracellular chromatin is often used to identify NETs in tissues (53, 54). As an example, citrullinated NETs were significantly associated with high histopathological tumor grades and lymph node metastasis in human CRC (**Figure 3**) (55).

### 2.4 Tumor cells induce NET formation

There are several reports documenting the presence of NETs in tumor tissues (56–58). In agreement with the co-cultivation of cancer cells with neutrophils resulting in NET formation within 3 hours, electron microscopy showed that neutrophils were destructed and did not provide evidence for DNA-containing vesicles budding from intact neutrophils (58). Moreover, the cancer cell-induced NET formation depended on NOX activity (58). Altogether these findings indicated that cancer cells induced suicidal rather than vital NET formation (58).

The TME is rich in factors that can promote NET formation from both TAN and granulocytic myeloid-derived suppressor cells (GR-MDSCs), such as granulocyte-colony-stimulating factor (G-CSF) and interleukin-8 (IL-8) (59–62). G-CSF is a cytokine produced by leukocytes, macrophages, endothelium, fibroblasts and cancer cells. The expression of G-CSF is highly increased in both murine and human tumor cells (63–65). G-CSF overexpression predisposes neutrophil recruitment into metastatic lesions and enhances migration and invasion of tumor cells *via* generation of ROS, NET formation and production of other pro-tumor proteins (59, 66–68). IL8 is a chemokine of the CXC glutamic acid-leucine-arginine motif bearing (ELR+) family and was initially identified as a powerful chemotactic factor for neutrophils (69, 70). IL8 is produced in large amounts by several human tumors and as a main agonist of CXCR1 and CXCR2 was mostly implicated in the recruitment of neutrophils and MDSCs (61, 71). In many types of human cancer, such as bladder cancer, non-small cell lung cancer (NSCLC) and metastatic melanoma NETs show a positive association with IL-8 in tumor tissues and serum (72). In patients with diffuse large B-cell lymphoma (DLBCL), DLBCL-derived IL8 interacting with CXCR2 on neutrophils resulted in NET formation *via* Src, p38 and ERK signalling. Blocking of the IL8–CXCR2 axis inhibited the formation of NETs (73). Conditioned media (CM) harvested from different cancer cell lines also induced NET formation, both from neutrophils and granulocytic myeloid-derived suppressor cells (GR-MDSCs). Blocking of CXCR1 and CXCR2 with Reparixin or a CXCR1 blocking monoclonal antibody (mAb) inhibited NET formation induced by the respective CM (61, 74). Besides G-CSF and IL-8, many additional pro-inflammatory cytokines and damage-associated molecular pattern (DAMP) present in the TIME are well established to promote NET formation. In consequence, it is generally accepted that the TIME plays a critical role in the development of malignant tumors and that NETs exhibit a significant impact on tumorigenesis (75). However, how the initiation and progression of tumors is regulated by NETs and how these functions are affected by different TIMEs remains elusive.

## 3 Crosstalk between NETs and adaptive TIME

In the following we will discuss the impact of different cell populations of the adaptive immune system on the formation and tumorigenic activities of NETs.

### 3.1 NETs cause exhaustion and dysfunction of CD8^+^ T cells

T cells are among the most plastic cells in response to different TIMEs as they can be rendered dysfunctional and exhausted by chronic persistent antigen stimulation allowing immune escape and augmenting tumor development (76). CD8^+^ T cells of the adaptive immune system are the most potent effectors in the anti-cancer immune response and serve as executors of cancer immunotherapies with a large impact on the outcome of many different tumors (77). An interplay between NETs and CD8^+^ T cells in the TIME was suggested by de Andrea and colleagues who reported that NET density in human tumor tissues and NET concentrations in the serum of cancer patients negatively correlated with CD8^+^ T cell counts in tumor tissues (72). In analogy, Kaltenmeier and colleagues reported that NET-rich TIMEs, both in a murine liver ischemia/reperfusion (IR) metastasis model and in a murine subcutaneous tumor model induced exhaustion and dysfunction of CD8^+^ T cells (78). This was accompanied by an increase of the exhaustion markers PD-1, Tim3, and Lag3 together with a diminished production of the effector cytokines IL-2, IFN-γ, and TNF-α, and altered metabolic profiles including decreased mitochondrial function, glucose uptake, and upregulated fatty acid intake (78). Continuous exposure of murine T cells to NETs *in vitro* induced an exhaustive phenotype as well, supporting direct effects of NETs on CD8^+^ T cells (78). The assessment of mechanisms how NETs regulate T cell function in the TIME identified that PD-L1 was embedded in the NET chromatin of wild type (WT) bone marrow (BM) derived-neutrophils treated with PMA (79). Immune inhibitory receptor PD-1 and its ligand PD-L1 were previously recognized as an immune inhibitory axis on the surface of T cells promoting the depletion of functional T cells and tumor immune escape (79). Accordingly, NETs may directly provoke the exhaustion of T cells *via* the PD-1/PD-L1 axis. Consequently, targeting of PD-L1 in NETs restored functional T cells and ameliorated the tumor burden (78).

Apart from direct effects of NETs on T cell exhaustion, NETs are also able to impair the cytotoxic function of T cells by physical shielding. Teijeira et al. identified in a co-culture system and a mouse model that NETs alone cannot impact tumor cell spheroid survival or proliferation (61). However, coating of tumor cells with NETs impaired cytotoxicity of CD8^+^ T cells and protected the tumor cells from direct contact with CTLs (61). Several other studies confirmed that NETs protect tumor cells by forming a physical barrier at the tumor/stroma interface. This abrogates the infiltration of CD8^+^ T cells into the tumor cell areas (58, 80, 81). Apparently, this barrier function of NETs can protect tumors from cytotoxic immune attacks.

### 3.2 NETs regulate the Th1/Th2 TIME

CD4^+^ T cells are essential regulators in cancer immunosurveillance. They modulate the tumor microenvironment and eradicate tumor cells. NETs can exert an immunosuppressive function by inhibition of CD4^+^ T cells (78). CD4^+^ T cells are highly heterogeneous and exert different immune responses to various pathogens (82). Three major subsets of CD4^+^ T cells have been identified so far: Th cells, Tregs and follicular helper T cells (TFH) (83). Th cells have drawn a lot of attention since their integral roles in the TIME was demonstrated by several pre-clinical and clinical models (84, 85). Th1 and Th2 are the two predominant categories of CD4^+^ Th cells. Th1 and Th2 are characterized by high levels of IFN-γ and IL-4 expression, respectively (86). For Th1 polarization, the signal transducer and activator of transcription-1 (STAT1) and STAT4 activated by IL-12 and IFN-γ induce the T-box transcription factor (T-bet) that drives Th1 differentiation and suppresses Th2 differentiation. IFN-γ in turn secreted from Th1 cells stabilizes Th1 differentiation forming a positive feedback loop (86, 87). Th2 differentiation is dependent on the expression of IL-4, which results in STAT6-mediated activation of the GATA3 transcription factor that stimulates Th2 polarization and suppresses Th1 differentiation. Similar to Th1 differentiation, an autocrine positive feedback loop of IL-4 stabilizes Th2 differentiation (87, 88). The balance between Th1 and Th2 CD4^+^ T cells is critically regulating tumorigenesis. A dominance of a Th1 and Th2 TIME is associated with improved or poor prognosis, respectively (88).

It has been shown that in some non-cancer diseases, such as type 1 diabetes (T1D), rheumatoid arthritis (RA), and chronic pneumonia NETs exacerbated Th1 responses by activation of dendritic cells (89–91). Moreover, NETs may also promote a Th1-like TIME through activation of peripheral blood mononuclear cells (PBMCs), which contributed to the recruitment of T cells and monocytes-macrophages and prevented tumor growth (92). In a murine model of bladder cancer Bacillus Calmette-Guerin (BCG) caused NET-induced apoptosis, cell-cycle arrest, and inhibited migration of tumor cells (92). In contrast, Zheng and colleagues found that in a murine breast cancer model with lung metastasis Th2 cytokines stimulated NET formation by maintaining complement 3 (C3) highly expressed in lung mesenchymal stromal cells (LMSCs) through the STAT6 signalling pathway (93). The Th2-STAT6-C3-NET cascade promoted lung metastasis and NET formation was essentially required for lung metastasis in the Th2 prominent TIME (93). Th2 cells are regarded as contributors to the immunosuppression and tumor escape, corresponding to NETs, which lead to the exhaustion and dysfunction of CLTs. The partially conflicting results indicate that different TIMEs and prognoses can be associated with NETs. However, it is clear that NETs can regulate the differentiation of TIME.

### 3.3 NETs influence Treg differentiation of CD4^+^ T cells

Th1 cells are the main cytotoxic subtype of CD4^+^ T cells. Tregs are critical for immune tolerance and homeostasis limiting potential collateral tissue damage after the initial CTL response. The central differentiation factor of Tregs is the transcription factor forkhead box protein P3 (FOXP3) (94). Wang and colleagues observed that in the non-alcoholic steatohepatitis-hepatocellular carcinoma model (NASH-HCC model, STAM) the overall number of CD4^+^ T cells dropped while the number of Tregs sharply increased coincident with more severe tumor burden. After depletion of Tregs by diphtheria toxin (DT) Th1 cells counts increased and tumor burden decreased (95). Meanwhile, it has been repeatedly confirmed that Tregs impair cancer immunosurveillance by suppressing immunity and hampering immune responses; that fosters tumor development and progression (96–99). Altogether, Tregs establish an immunosuppressive subset of CD4^+^ T cells and play an important role in the TIME. Interestingly, in murine models of liver cancer [non-alcoholic fatty liver disease (NAFLD), non-alcoholic steatohepatitis (NASH)] NETs co-localized with Tregs, occurred in early stages, and persisted during cancer development (95). Also in humans a positive correlation between the presence of the NET marker H3cit and FoxP3 was observed in livers of patients with NASH or NASH-HCC (95). From this it has been suggested that Tregs may induce NET formation (95). Vice versa, NETs can also induce Treg differentiation from naïve CD4^+^ T cells in the NASH liver microenvironment through the reprogramming of metabolic pathways involved in the oxidative phosphorylation (OXPHOS) of mitochondrial respiration (95). NETs activated TLR4 on the surface of naïve CD4^+^ T cells and subsequently modulated OXPHOS by enhancing oxidation of NADH to NAD+, which was required for Treg differentiation (95, 100). This was further confirmed by NET ablation, which was associated with a decrease of Treg-specific FoxP3 protein levels in the liver of STAM mice followed by alleviated HCC development and progression (95).

In conclusion, most studies summarized in the chapter above indicate that NETs exert immunosuppressive functions on CD8^+^ T cells and CD4^+^ T cells augmenting the progression of tumors, further supporting an important role of NETs in the TIME. Enigmatically, in certain non-cancer diseases NETs appear pro-inflammatory through eliciting immune functions.

## 4 Crosstalk between NETs and innate TIME

In the following we will discuss the impact of cell subtypes of the innate immune system on the formation and tumorigenic activities of NETs.

### 4.1 Interactions between NETs and macrophages

The innate immunity establishes the first line of anti-microbial defence with a major impact on the TIME. Various kinds of macrophages together with dendritic cells, histiocytes, Kupffer cells, and mast cells are the gate keepers of innate immunity. Besides long-lived sessile tissue macrophages, monocytes from peripheral blood differentiate into macrophages after recruitment to tissues. Macrophages make up the majority of myeloid immune cells in the TIME (101, 102). They possess phagocytic functions and serve as antigen presenters in the TIME. Dependent on their activation, macrophages can be polarized into many distinct subsets, with M1 and M2 macrophages being the extremes of a multidimensional continuum (29).

M1 macrophages are induced in a Th1 TIME by pro-inflammatory cytokines, such as IFN-γ. They mediate the defence of the host from a variety of antigens and have roles in the anti-tumor immunity (103). M2 macrophages are induced in a Th2 TIME and exert an anti-inflammatory function and immunosuppressive activity (103).

Beyond their antigen-presenting capabilities and phagocytic functions, macrophages regulate NET formation and clearance. Haider and colleagues analysed abdominal aortic aneurysm (AAA) and found macrophage counts to be inversely correlated with NETs in both the intraluminal thrombi and the vessel wall (104). Macrophages, when seeded on blood-clots coated by NETs *in vitro* showed the ability to digest NETs by secreted deoxyribonucleases (DNases) followed by phagocytosis of the NET remnants (104). Specially, M1 macrophages were most active in NET clearance by macropinocytosis (104). Expanding on macrophage-dependent NET degradation, Fadeel and colleagues reported that physiological concentrations of extracellular DNase I were not sufficient to completely clear NETs and the cytosolic exonuclease TREX 1 (DNase III) in macrophages was required for the digestion of NETs after phagocytic uptake (105, 106).

In contrast, other reports indicated that macrophages can also induce the formation of NETs. It was shown that exosomal miR-146a from oxidized low-density lipoprotein (oxLDL)-stimulated macrophages promoted the excessive release of NETs, concomitant with the overexpression of ROS and high levels of pro-inflammatory cytokines such as IL-8, TNF-α, IL-6, and IL-1β (107). This indicated that according to their stimulation macrophages may decrease or increase the concentrations of NETs in tissues. In a third scenario macrophages may also mediate the activity of NETs. For example in an *in vitro* co-culture system of macrophages and A549 lung cancer cells, Zhang and colleagues reported that addition of purified NETs promoted migration and invasion of A549 lung cancer cells (108). They suggested that this partly depended on the cytokines IL-1β, IL-6, IL-18 and TNF-α, which were released from the macrophages (108). Interestingly, a subsequent study showed that the treatment of macrophages with NETs also increased extracellular M1 macrophage-derived DNA, likely representing METs (109). Accordingly, METs may have contributed to the activation of migration and invasion of tumor cells in the co-culture experiment.

In conclusion, it is best established that macrophages exert disintegrating functions on NETs. However, sporadic reports on different effects of macrophages on the formation and the activity of NETs indicate that the specific outcome of the interaction of NETs with macrophages may be TIME related, warranting further investigations.

### 4.2 METs and NETs

Not only neutrophils but also macrophages can form net-like structures, referred to as METs. Similar to NETs, METs are composed of DNA and extracellular proteins and are produced in response to various pathogens and chemical stimuli. METs are generated in the course of a unique cell death program of macrophages (METosis) and until now have only been observed in the inflammatory M1 subset after treatment with IFN-γ and LPS (109, 110).

Only few differences have been reported between NETs and METs. King and colleagues noted at the morphological level that chromatin fragments are longer in NETs as compared to METs (111). In addition, METs are associated with the macrophage marker protein CD68 and the matrix metalloproteinase (MMP) 12 (111–113). Moreover, differences have been reported at the signalling processes responsible for extracellular trap formation. For example, Aulik and colleagues reported that after stimulation of cells MET formation occurs faster than NET formation (114). Finally, PAD4 and PAD2 are considered to be the main promoters of NET and MET formation, respectively (115, 116). Notably, elastase and MPO, which are often regarded as neutrophil-specific markers, have also been identified in METs (117, 118). Accordingly, co-staining of H3cit together with CD68 seems to be at present the most specific approach to distinguish METs and NETs (111–113). The differences between NETs and METs may cause their different roles in host defence.

NETs and METs generally protect against microorganisms by immobilizing them (119). However, at present it is not clear whether this immobilization is specifically microbicidal, because MET formation has also been found to promote pathogen growth by providing a scaffold for aggregation rather than killing pathogens (114, 119, 120).

In tumor tissues, macrophages are often present in higher numbers as compared to neutrophils (121). However, the presence of METs and NETs was not found to be significantly related with the infiltration of the respective cell types (122). Accordingly, the presence of METs and NETs may be more strongly related to the responsiveness of the respective cell types to TIME-derived stimuli and to different stability of NETs and METs. The first hypothesis was supported by the fact that higher concentrations of PMA are required to induce the formation of METs compared with NETs and that this process is accompanied by a higher ROS production in macrophages (111, 114).

To specifically address the relevance of METs in cancers, a retrospective study of 135 patients after radical resection of non-functioning pancreatic neuroendocrine tumors (pNETs) was analysed and showed that both NETs and METs were associated with shorter recurrence-free survival (RFS) (122). In univariate and multivariate Cox regression analyses NETs and METs were independent prognostic factors for shorter RFS and better indicators than WHO grade and TNM stage to predict the prognosis for patients (122). This finding was confirmed in another study with 116 CRC patients. In a training and a validation cohort (n=94), METs were identified as an independent prognostic factor to predict the 5-year overall survival rate in CRC and significantly associated with distant metastasis but not with local tumor invasion and lymph node metastasis (35).

Co-cultivation of the CRC tumor cell lines HCT-116 and SW480 with macrophages and a neutrophil-deficient mouse model were used to investigate the crosstalk between METs and CRC cell lines. These studies revealed that METs in the absence of NETs can activate the migration and invasion of CRC tumor cells and promote liver metastases (35). For example, the inhibition of PAD2 impeded MET formation from macrophages, blocked the crosstalk between CRC cells and METs and reduced liver metastasis (35). From this it can be concluded that targeting of both METs and NETs expands the palette of new therapeutic targets for cancer patients.

### 4.3 NETs impair the anti-tumor effects of NK cells

NK cells are innate cytotoxic lymphoid cells acting against tumor cells and pathogens. Activated NK cells exert their cytotoxic functions by releasing perforin and granzymes. They secrete cytokines, such as IFN-γ that participate in the orchestration of the adaptive immune response (123, 124). NK cells, unlike CTLs, are not restricted by the major histocompatibility complex (MHC). They can identify and destroy tumor cells without exposing tumor-specific antigens. This considerably accelerates the anti-cancer immune response (125, 126). The interaction between neutrophils and NK cells has emerged as an important mechanism for the modulation of immunological responses (127). NK cell-derived IFN-γ, GM-CSF and TNF-α not only prolong neutrophil survival and induce neutrophil activation, but are also essential for NET formation (128, 129). Of note, surgery promotes the production of fibrin and platelet clots coating tumor cell emboli. This limits NK cell-mediated tumor clearance (130, 131). In addition, surgery was found to stimulate NET formation and exacerbated distal organ injury by the activation of a systemic procoagulant state and diffuse microvascular immune thrombi (132–134). Clinical trials showed convincing therapeutic effects of NK cell infusion in various hematological malignancies (135). However, NET-coated tumor spheroids protected tumor cells from cytotoxicity of NK cells (61). *In vitro*, NETs inhibited migration and motility of NK cells indicating a direct effect of NETs (61). In a NET-rich TIME the therapeutic efficiency of NK cells was clearly impaired (135). Moreover, the inhibition of NETs in a murine model of HCC enhanced the anti-tumor immunity mediated by NK cells (135). Besides physical shielding tumor cells from attacks of NK cells, further mechanisms may exist that impair the anti-tumor function of NK cells. For example, NETs may activate platelets to impair the NK cell mediated tumorilytic function by increasing the secretion of transforming growth factor (TGF)-β, which can inhibit the mobilization of NK cells (136, 137). MMP-9 in NETs may also be implicated in NK cell dysfunction resulting in tumor immune evasion (138). All of these results indicate a mutual interaction of NETs and NK cells, which in an inflammatory TIME fosters the tumorigenic functions of NETs by counteracting NK cell activity.

### 4.4 DCs present antigens from NETs

Although DCs constitute a rare immune cell population in tumors and lymphoid organs, these cells are crucial for specialized antigen-presentation, the regulation of innate immunity, and the initiation of the adaptive immune response (129, 139). DCs as antigen-presenting cells are required for T cell responses and are widely used in vaccination. DCs include several subsets, such as plasmacytoid DCs (pDCs), conventional DCs (cDCs) [also referred to as myeloid DCs (mDCs)], and monocyte-derived DCs (moDCs). cDCs are the major DC population and they play a critical role in anti-tumor responses. They endocytose apoptotic and necrotic tumor cells and present tumor-related antigens to T cells (140). This may happen either in an activating or a tolerogenic manner. While pDCs mainly produce large amounts of IFN-α and IFN-β and can also be stimulated to directly activate T cells (141, 142). In patients with psoriasis and systemic lupus erythematosus NETs modulate the crosstalk between innate and adaptive immune responses and activate pDCs *via* TLR9 (143, 144). Sangaletti and colleagues described co-cultures of mDCs with inflammatory polymorphonuclear leukocytes (PMNs) were prone for NET formation, which induced a stable interaction of NETs and mDCs, subsequently resulting in mDCs loaded with NET components, including extracellular proteins and DNA (145). Thus, mDCs were capable to take up antigens of NETs for potential antigen processing and presentation (145). *In vivo*, immunization with mDCs co-cultured with NETs or apoptotic/necrotic PMNs induced anti-neutrophil cytoplasmic antibodies (ANCAs) and anti-dsDNA autoantibodies, while mDCs or NETs alone had no effect (145). Grippingly, ANCA-related autoimmune vasculitis was exclusively found in the renal and pulmonary parenchyma of mice inoculated by NET-loaded mDCs, although measurable amounts of ANCA were also detected in mice inoculated by mDCs co-cultured with apoptotic/secondarily necrotic PMNs (145). This indicated that the structural integrity of NETs was required for the transfer of cytoplasmic neutrophil antigens to mDCs (145). The autoimmune features observed in immunized mice were shared by human autoimmune systemic vasculitis (145). The interaction between NETs and mDCs might lead to autoimmunity and underlies the dynamics of ANCA induction in humans.

In cancer DCs acquire, process and present tumor-associated antigens (TAAs) on MHC molecules and shape adaptive immune responses (141). However, the investigation of the interaction of NETs and DCs in tumors is still at the beginning. Tripodo and colleagues described that NETs can boost DC vaccination against acute myeloid leukemia (AML) (146). They established a h-MRP8-NPM1*+* (NPMc*+*) transgenic mouse model, which developed myeloproliferation without inducing overt AML (146). DCs co-cultured with NETs from NPMc+ mice were used to treat NPMc+ mice, thereby reducing NPMc+ myeloproliferation with a deceleration of myeloid expansion and a reduction of myeloid blasts by triggering immune activation (146). To further evaluate the impact of DCs uploaded with NPMc+ NETs on AML, NPMc+ mice were implanted subcutaneously with the leukemia cell line C1498 with mutant NPM1 (C1498-NPMc+) followed by a treatment with NPMc+ NETs/DC vaccine. A reduced tumor growth and stronger CTL cytotoxicity against NPMc was observed (146). NPMc+ NETs/DC vaccination enhanced anti-tumor immunity and prevented growth of leukemia transplants (146). These data indicated that NETs improved anti-tumor vaccination and tumor antigens trapped by NETs could be used to boost immune responses to cancer vaccines.

## 5 NETs and Immunotherapy

The studies above provided new insights into the role of NETs in the TIME. Currently, it is established that under certain conditions NETs foster immunosuppression and abrogate the efficacy of immunotherapy. Zhang and colleagues reported that in pancreatic ductal adenocarcinomas (PDAC), neutrophils were recruited by IL-17, subsequently released NETs, and thereby induced immunosuppression (147). This was attributed to the activation of immune checkpoints, depletion of CTLs, and direct protective functions of NETs that protected tumor cells from cytotoxic attack (147). The deletion of NETs together with the application of immune checkpoint inhibition fostered anti-tumorigenic responses in the PDAC mouse model dramatically as compared to the treatment with immune checkpoint inhibition alone (147). This indicated that the removal of NETs might overcome the resistance of immune checkpoint inhibitors and restore adaptive immune responses in certain conditions. Similarly, Zhang and colleagues reported that the degradation of NETs with DNase I highly increased therapeutic benefits of anti-PD-1 immune treatment in a MC38-bearing mouse model of CRC (148). Mechanistically, the combination of DNase I and PD-1 inhibition resulted in the increased CD8^+^ T cell infiltration and cytotoxicity, eventually overcoming the resistance to anti-PD-1 monotherapy (148). However, considering its short biologic half-life, repeated daily injections of DNase I that are required to ensure an adequate drug level limits its therapeutic application. To overcome this limitation Xia et al. created an adeno-associated virus (AAV) gene therapy vector that expressed DNase I selectively in the liver as potential anti-tumor therapy (149). In this study, a single intravenous injection of AAV maintained sufficient long-term hepatic expression of DNase I (149). In a corresponding murine model CD8^+^ T cell counts and local immune responses were restored in the TIME, and the development of liver metastases was suppressed (149).

In addition to adaptive immune therapy, innate immune therapy also has been advocated as an appealing option for cancer treatment. NK cell infusion is considered as a potential option in cancer therapy. However, the acidic TIME and NETs severely counteract its efficacy (135). Cheng at el. designed an *in situ* injectable dual pH-responsive adhesive hemostatic hydrogel that neutralized tumor acidity and digested NETs to improve the therapeutic efficiency of adoptive NK cells for the prevention of HCC recurrence after surgery (135). In more detail, they applied biocompatible mesoporous bioactive glass nanoparticles (MBGNs) loaded with DNase I. Subsequently, the nanoparticles were incorporated into a hydrogel (GODM-gel). Injection of this GODM-gel into liver resection margins in the orthotopic HCC murine model neutralized tumor acidity, destructed NETs and significantly augmented NK cell infiltration and prevented HCC recurrence after surgery (135). These findings indicate that NETs in the TIME are a promising target to improve immune checkpoint therapy and to avoid disease recurrence after tumor surgery.

## 6 Conclusions

According to the available data, the tight relationship between NETs, tumor cells, and TIME sheds light on the pivotal function of NETs in cancer progression and metastasis. In most cancer related diseases, NETs emerge as villains that drive metastasis by suppressing innate and adaptive immune responses. Immune therapies combined with the targeting of NETs was proven to enhance anti-tumor efficacy and to reduce drug resistance, providing new therapeutic strategies for patients with cancer. It is necessary to further elucidate the crosstalk between NETs, other extracellular traps and TIME. **Table 1** summarizes some key questions in this effort. Based on these open questions more studies are warranted to implement novel pharmacological interventions that target NETs.

**Table 1:** Future questions on the role of extracellular traps in tumorigenesis.

1	Which molecular components of NETs mediate their tumorigenic functions?
2	Do NETs cooperate with other extracellular traps (e.g. METs) in tumorigenesis?
3	Does the TIME modulate the activity of NETs?
4	What is the impact of extracellular traps on the local immune environment?
5	Will combined targeting of NETs and other extracellular traps reveal increased therapeutic efficacy in cancers?
6	What are the side effects and limitations of NETs/extracellular traps-targeted therapies against cancers?

## Author contributions

Author contributions: QF, MH and MS elaborated the subject of the review. QF wrote the manuscript. AS, EN, JK, MH and MS helped writing, provided helpful ideas and corrected the manuscript. All authors contributed to the article and approved the submitted version.
